# Author Correction: Comprehensive integrative analyses identify *GLT8D1* and *CSNK2B* as schizophrenia risk genes

**DOI:** 10.1038/s41467-018-07401-9

**Published:** 2018-11-16

**Authors:** Cui-Ping Yang, Xiaoyan Li, Yong Wu, Qiushuo Shen, Yong Zeng, Qiuxia Xiong, Mengping Wei, Chunhui Chen, Jiewei Liu, Yongxia Huo, Kaiqin Li, Gui Xue, Yong-Gang Yao, Chen Zhang, Ming Li, Yongbin Chen, Xiong-Jian Luo

**Affiliations:** 1 Key Laboratory of Animal Models and Human Disease Mechanisms of the Chinese Academy of Sciences and Yunnan Province, Kunming Institute of Zoology, Chinese Academy of Sciences, 650223 Kunming, Yunnan China; 2 Kunming College of Life Science, University of Chinese Academy of Sciences, Kunming, 650204 China; 3 Department of Psychiatry, The First Affiliated Hospital of Kunming Medical College, 650031 Kunming, Yunnan China; 4 State Key Laboratory of Membrane Biology, PKU-IDG/McGovern Institute for Brain Research, School of Life Sciences, Peking University, 100871 Beijing, China; 5 State Key Laboratory of Cognitive Neuroscience and Learning, and IDG/McGovern Institute for Brain Research, Beijing Normal University, 100875 Beijing, China; 6 CAS Center for Excellence in Brain Science and Intelligence Technology, Chinese Academy of Sciences, 200031 Shanghai, China; 7 Center for Excellence in Animal Evolution and Genetics, Chinese Academy of Sciences, 650223 Kunming, Yunnna China

Correction to: *Nature Communications* 10.1038/s41467-018-03247-3; published online 26 February 2018

In the original version of this Article, the affiliation details for Qiushuo Shen incorrectly omitted ‘Kunming College of Life Science, University of Chinese Academy of Sciences, Kunming, 650204, China’. This has now been corrected in both the PDF and HTML versions of the Article.

