# Supplementary material for: Comprehensive integrative analyses identify GLT8D1 and CSNK2B as schizophrenia risk genes
Source: Nat Commun. 2018 Feb 26;9:838. doi: 10.1038/s41467-018-03247-3 (PMC5826945; doi:10.1038/s41467-018-03247-3)
Supplement: Supplementary file 2 — Supplementary Information [file 41467_2018_3247_MOESM2_ESM.pdf]

# **Comprehensive integrative analyses identify *GLT8D1* and *CSNK2B* as schizophrenia risk genes**

Yang et al.

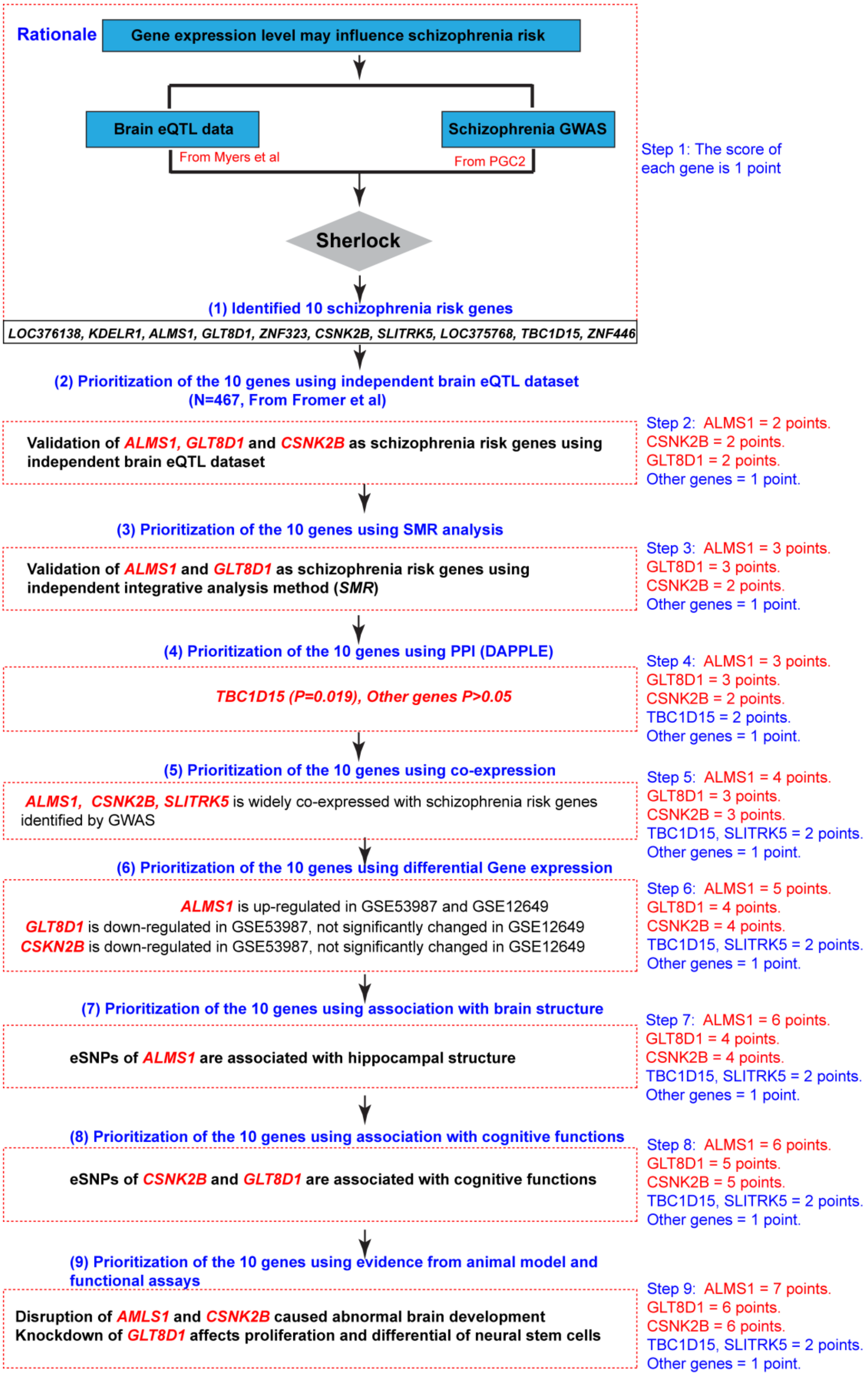


**Supplementary Figure 1. Prioritization of the genes identified by *Sherlock* integrative analysis using convergent functional genomics.** Evidence from each analysis contributes one

point to the studied genes in CFG analysis and a cumulative scoring strategy was used to calculate the final score of the studied genes. CFG analysis suggested that *AMLS1*, *GLT8D1* and *CSNK2B* may represent promising schizophrenia risk genes.

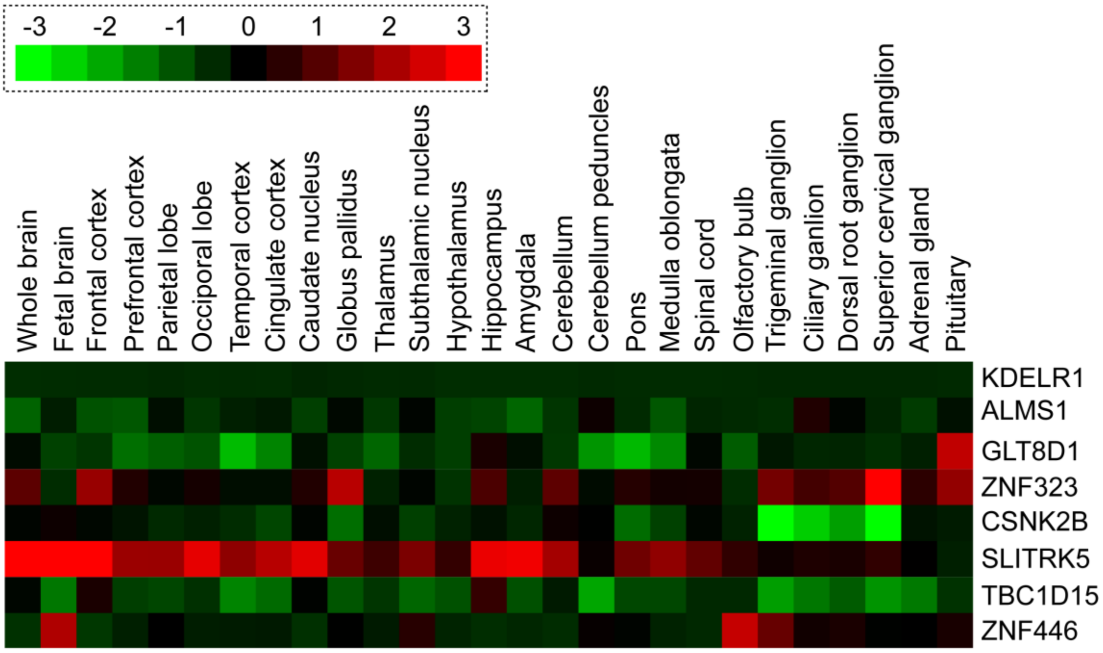


**Supplementary Figure 2. Top genes identified by *Sherlock* integrative analysis are widely expressed in human brain. The profiling was based on data from Benita et al[1].**

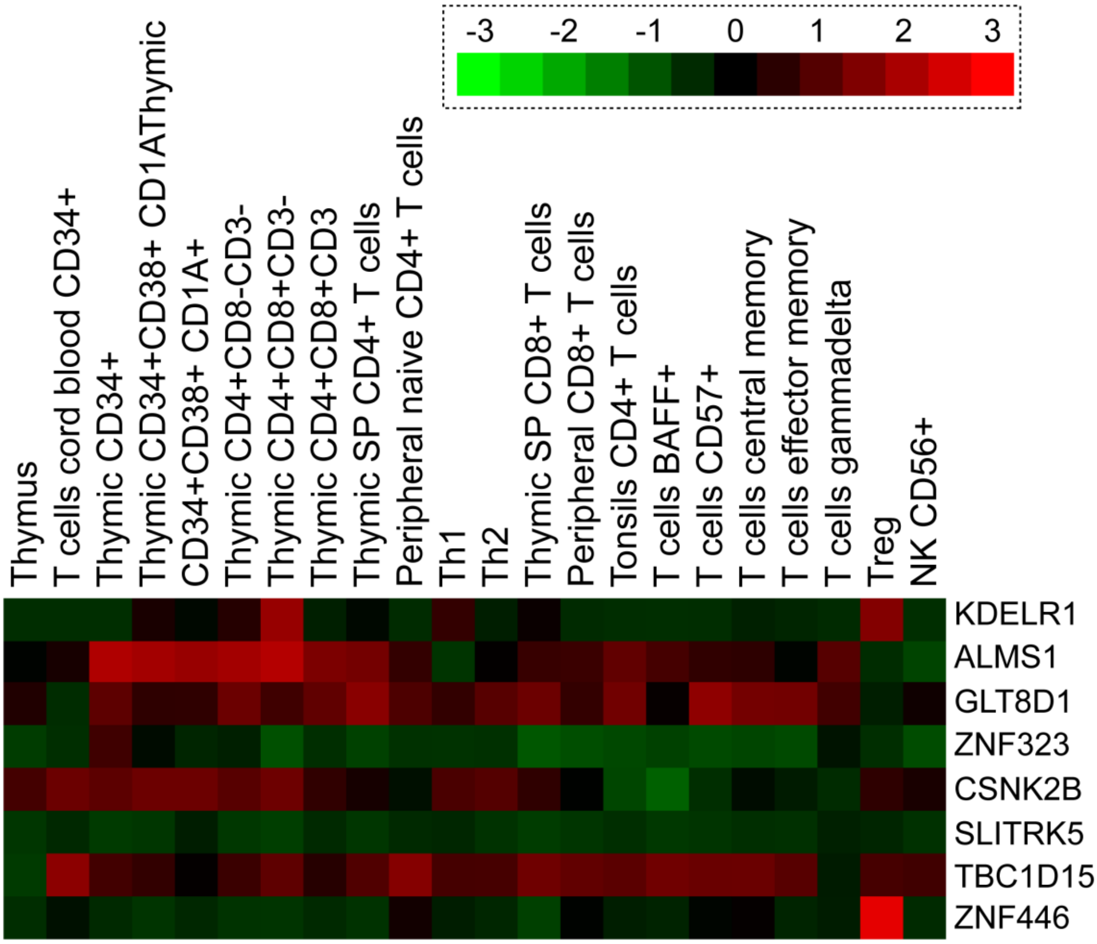


**Supplementary Figure 3. Top genes identified by *Sherlock* integrative analysis are widely expressed in human immune system. The profiling was based on data from Benita et al[1].**

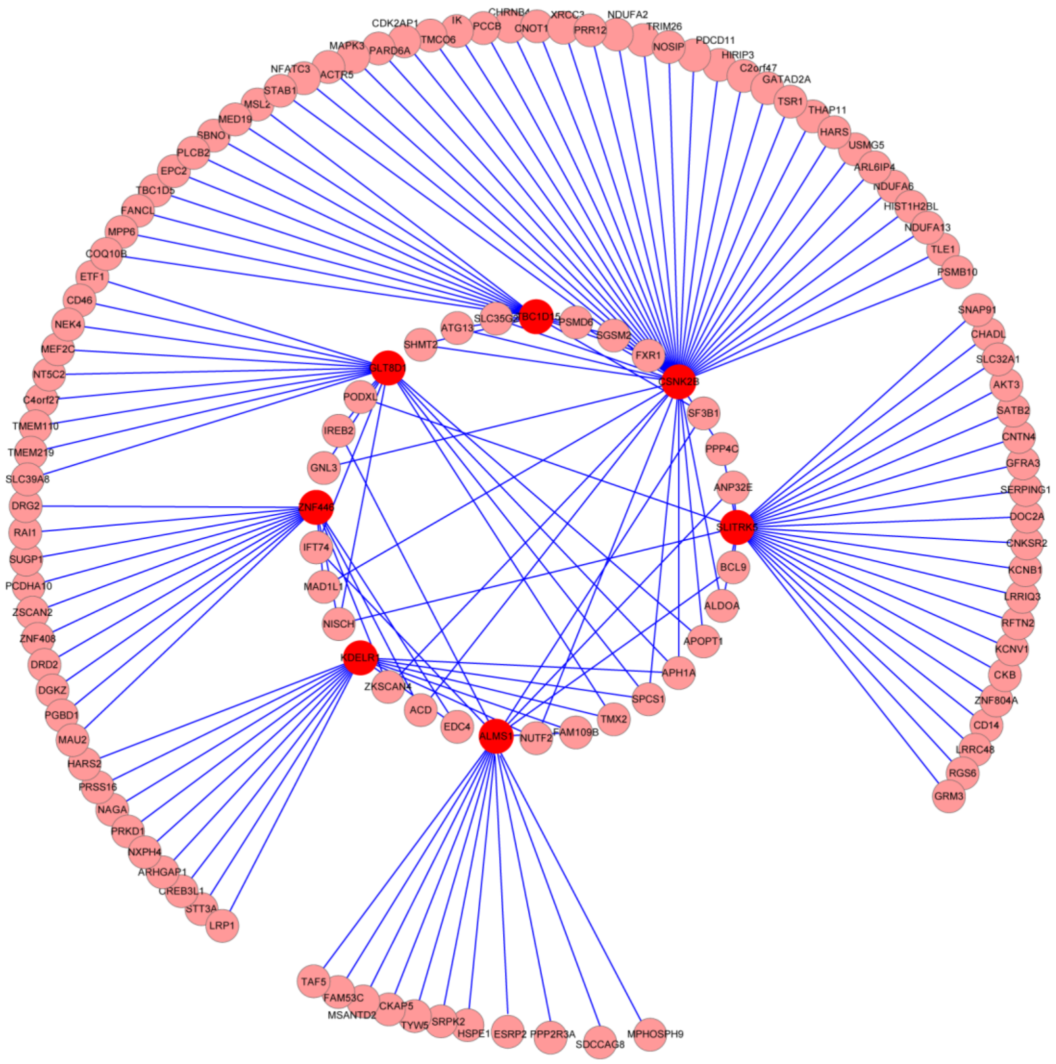


**Supplementary Figure 4. Protein-protein interaction between proteins encoded by schizophrenia risk genes identified by *Sherlock* and GWAS.** Proteins encoded by risk genes identified by *Sherlock* in this study are significantly interacted (permutation *P*=0.045) with proteins encoded by genes identified by previous GWAS of schizophrenia. Among the 8 top genes predicted by *Sherlock*, *CSNK2B* has the most interactions.

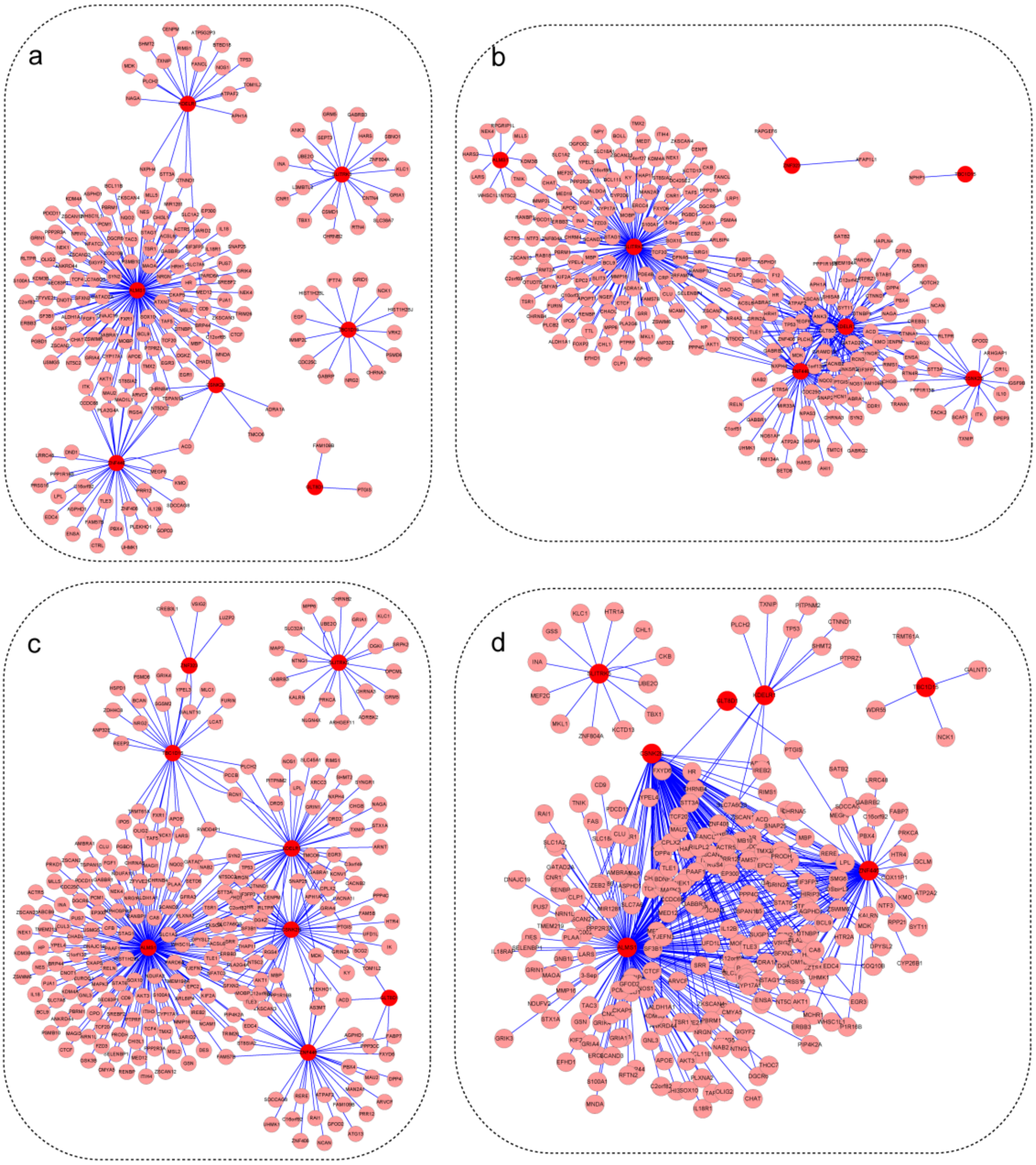


**Supplementary Figure 5. Co-expression network of risk genes identified by *Sherlock* integrative analysis and GWAS.** Genes identified by *Sherlock* in this study and previous schizophrenia GWAS showed distinct co-expression in different brain regions. Genes identified by *Sherlock* are significantly co-expressed with genome-wide significant schizophrenia risk genes in the MD-CBC cluster ($P<1.0\times10^{-3}$) (**b**), STR-AMY cluster ($P=1.2\times10^{-2}$) (**c**) and V1C-STC cluster ($P=4\times10^{-3}$) (**d**). *ALMS1* has the most connections (i.e., co-expressed with schizophrenia susceptibility genes identified by GWAS) in the PFC-MSC cluster (**a**), STR-AMY cluster (**c**), and V1C-STC cluster (**d**). In the MD-CBC cluster, *SLITRK5* has the most number of connections (**b**). The expression data were taken from the BrainSpan[2].

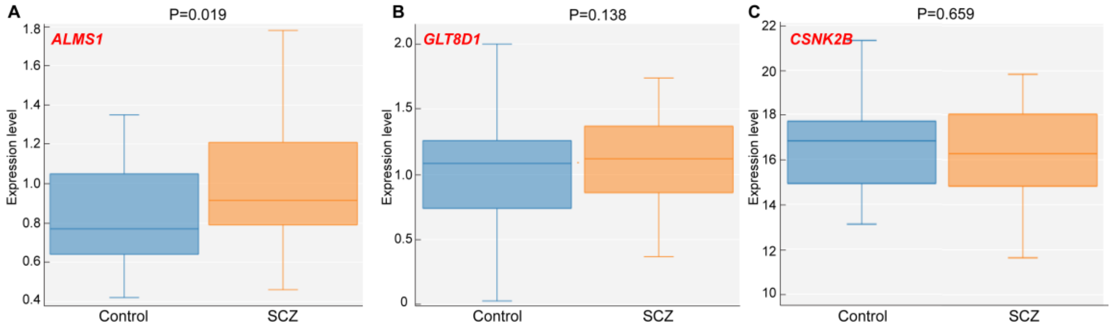


**Supplementary Figure 6. Expression of *ALMS1*, *GLT8D1* and *CSNK2B* in schizophrenia cases (SCZ) and healthy controls (Control).** *ALMS1* is significantly up-regulated in the prefrontal cortex of schizophrenia cases compared with healthy controls. (Data from GSE12649, including 35 schizophrenia cases and 34 healthy controls[3]).

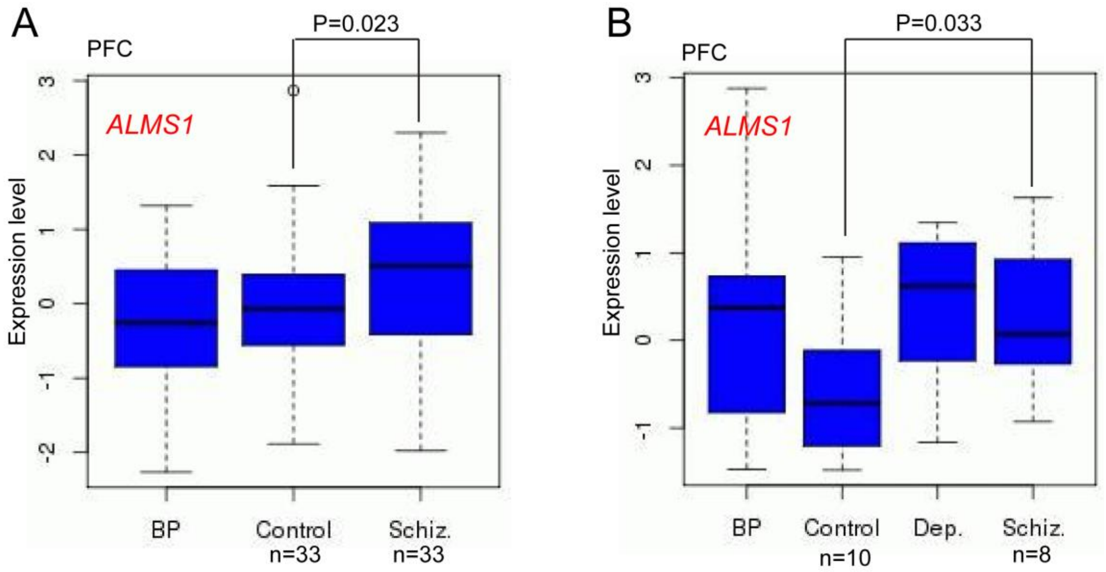


**Supplementary Figure 7. Expression of *ALMS1* is significantly up-regulated in the prefrontal cortex of schizophrenia cases compared with healthy controls** (Data from the Stanley neuropathology consortium integrative database[4]). BP, bipolar disorder; Schiz, schizophrenia cases; Control, healthy subjects.

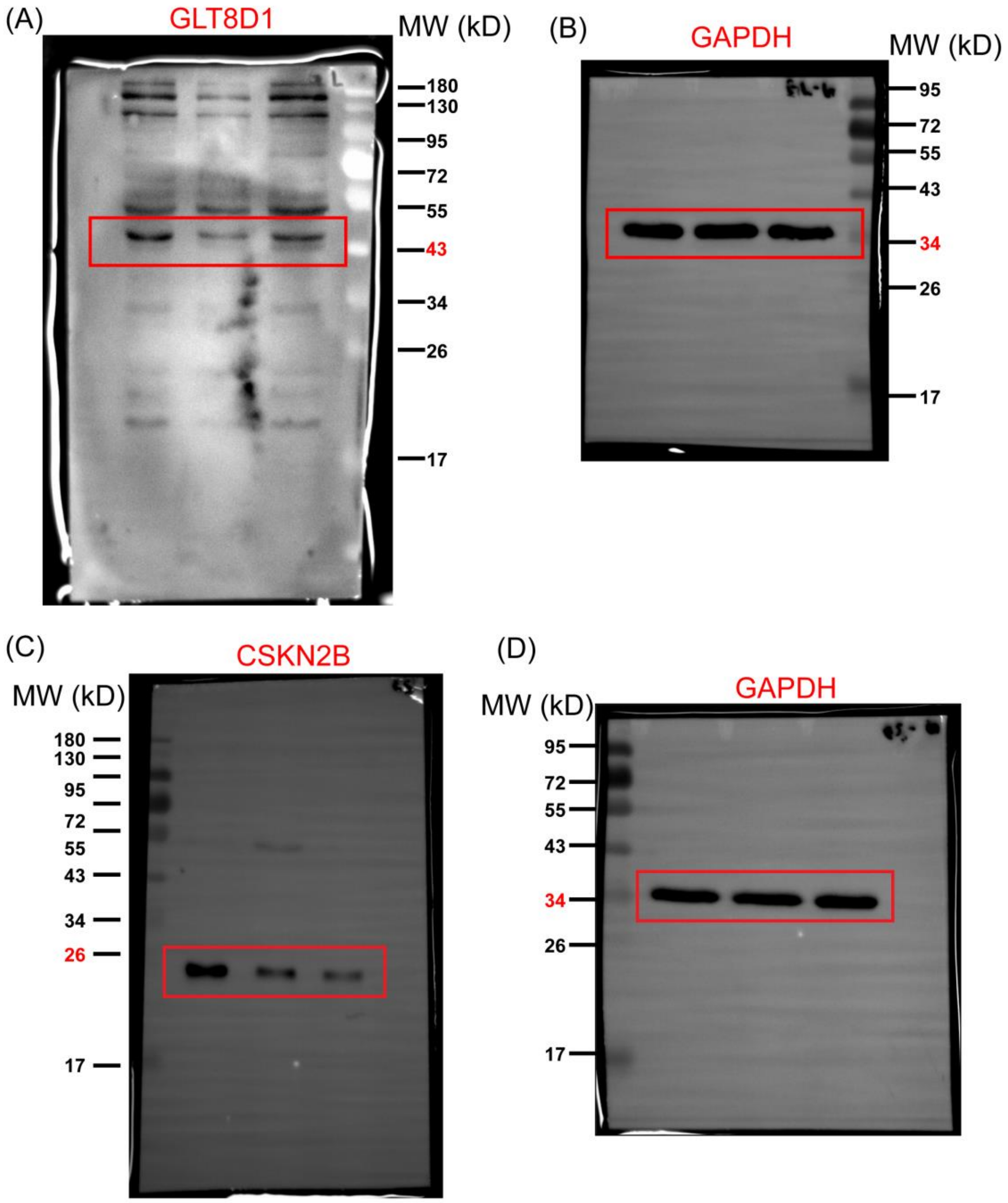


**Supplementary Figure 8. Uncropped western blot results shown in Figure 4b.** Blots marked by red box were shown in Figure 4b. MW, molecular weight. The GLT8D1 protein has 371 amino acids and CSNK2B has 236 amino acids.

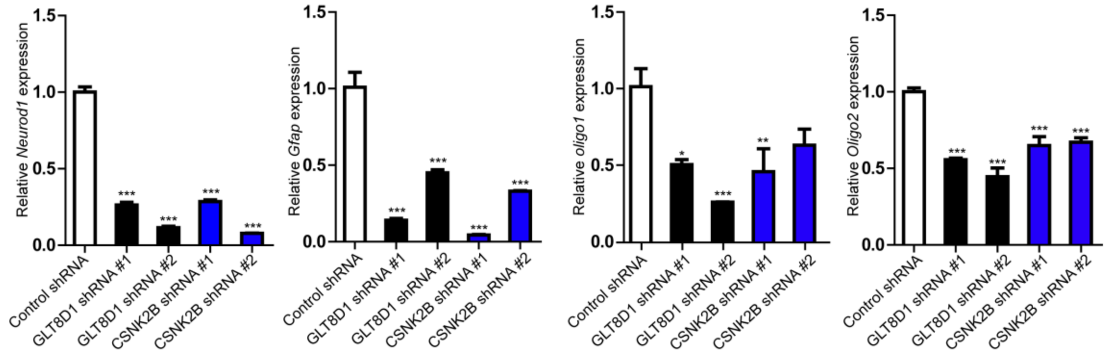


**Supplementary Figure 9. *GLT8D1* and *CSNK2B* knockdown significantly reduced the expression of differentiation related genes (*Neurod1, Gfap, Oligo1 and Oligo2*) compared with control groups. *GAPDH* was used as the internal control.** n=3. Data represent mean±s.d. **P*<0.05, ***P*<0.01, *** *P*<0.001, *Student's t*-test.

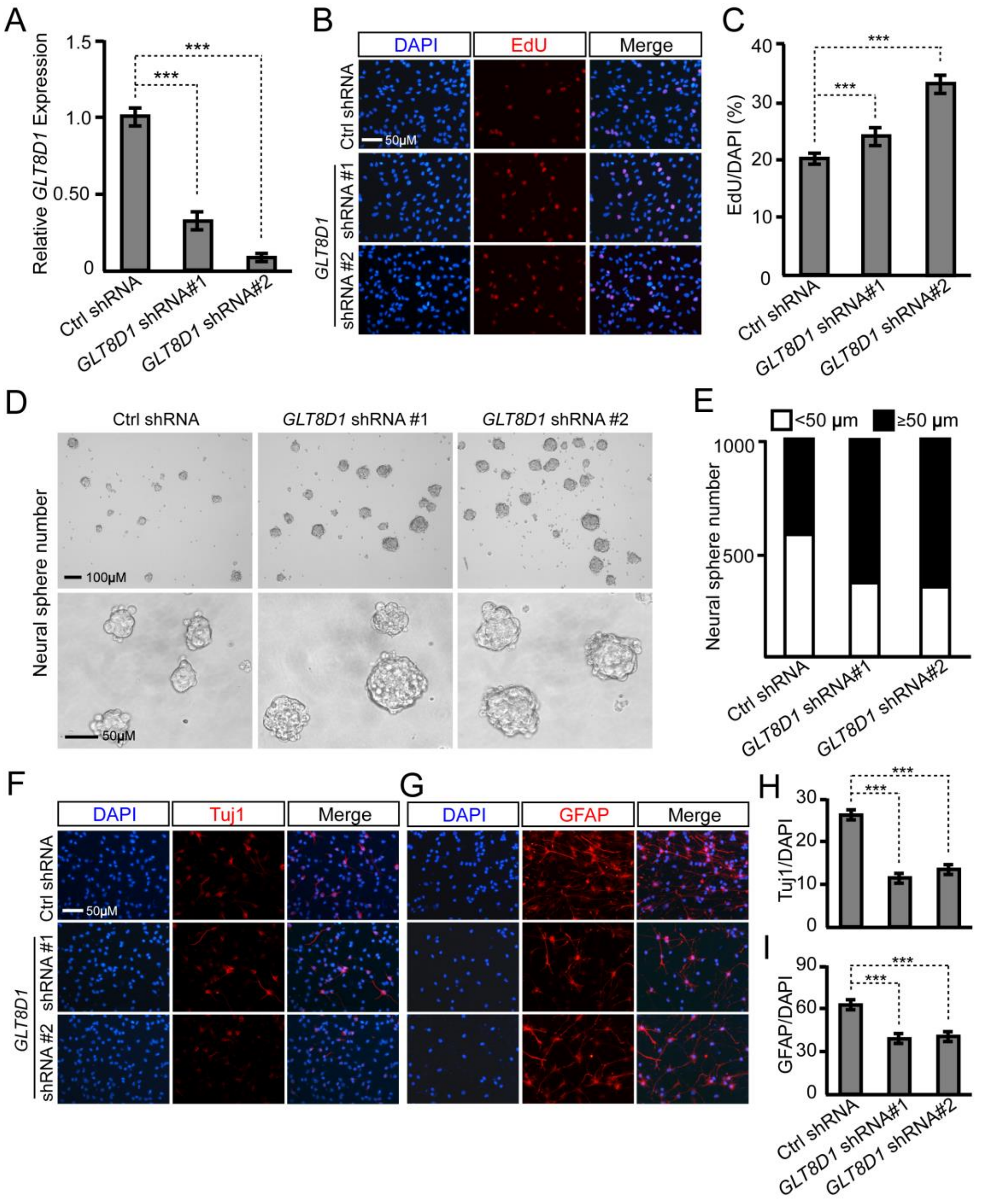


**Supplementary Figure 10. *GLT8D1* regulates the proliferation, self-renew and differentiation of neural stem cells.** NSCs were isolated from P7 dentate gyrus. **(A)** Individual *GLT8D1* shRNA knockdown efficiency was verified by real-time PCR. Ctrl: scramble shRNA control; shRNA #1 and shRNA #2 were used for *GLT8D1* knockdown. **(B)** EdU labeling revealed that *GLT8D1* knockdown enhanced proliferation of NSCs. **(C)** Quantification data for (B). **(D,E)** *GLT8D1* knockdown promotes self-renew ability of NSCs. Representative NSC neurosphere cultures and the quantification data were showed. **(F-I)** Differentiation of NSCs was impaired in *GLT8D1* knockdown NSCs compared with controls. Tuj1 (a marker for mature neurons) and GFAP (a marker for glia cells) were used to label differentiated mature neurons and glia cells, respectively. DAPI was used to label nuclei. All of experiments were performed for three independent biological assays (n=3) with at least three technique replicates, and had similar results.

For qPCR, *GAPDH* was used as the internal control. Data represent mean±s.d. Two-tailed *Student's* t-test was used to test if the difference reached significance level (0.05). ***$P$<0.001.

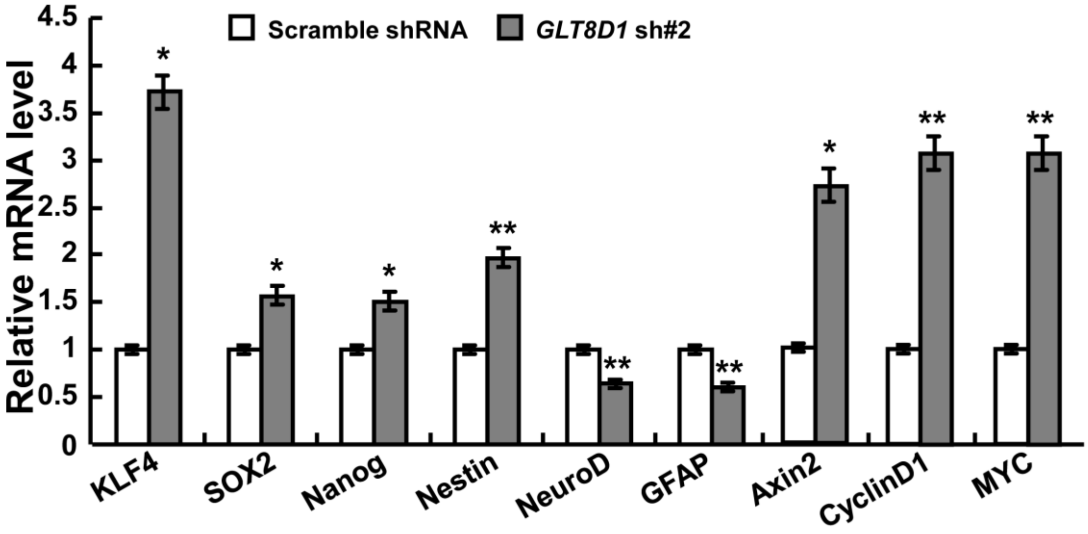


**Supplementary Figure 11. *GLT8D1* knockdown dysregulates multiple gene expressions which are important for NSCs self-renew and differentiation.** The expression of indicated genes was determined by qPCR and normalized to *GAPDH* expression. All of experiments were performed for three independent biological assays (n=3) with at least three technique replicates. Data represent mean±s.d. *$P$<0.05, **$P$<0.01, *t*-test.

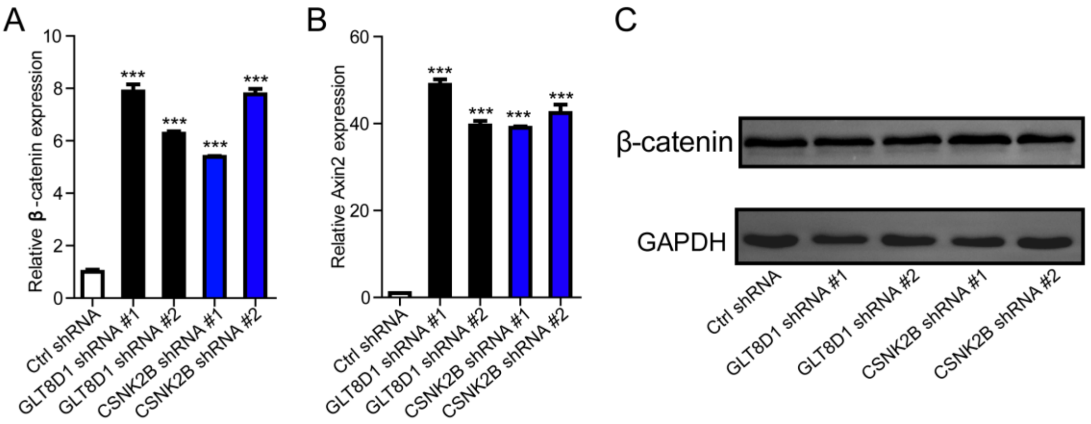


**Supplementary Figure 12.** (A, B) Expression of *β-catenin* and *Axin2* (genes involved in Wnt signaling pathway) was significantly up-regulated in *GLT8D1* and *CSNK2B* knockdown NSCs. (C) Protein level of β-catenin was not changed in *GLT8D1* knockdown NSCs and slightly up-regulated in *CSNK2B* knockdown NSCs. All of experiments were performed for three independent biological assays (n=3) with at least three technique replicates. *GAPDH* was used as the internal control. Data represent mean±s.d. ***$P$<0.001, *t*-test.

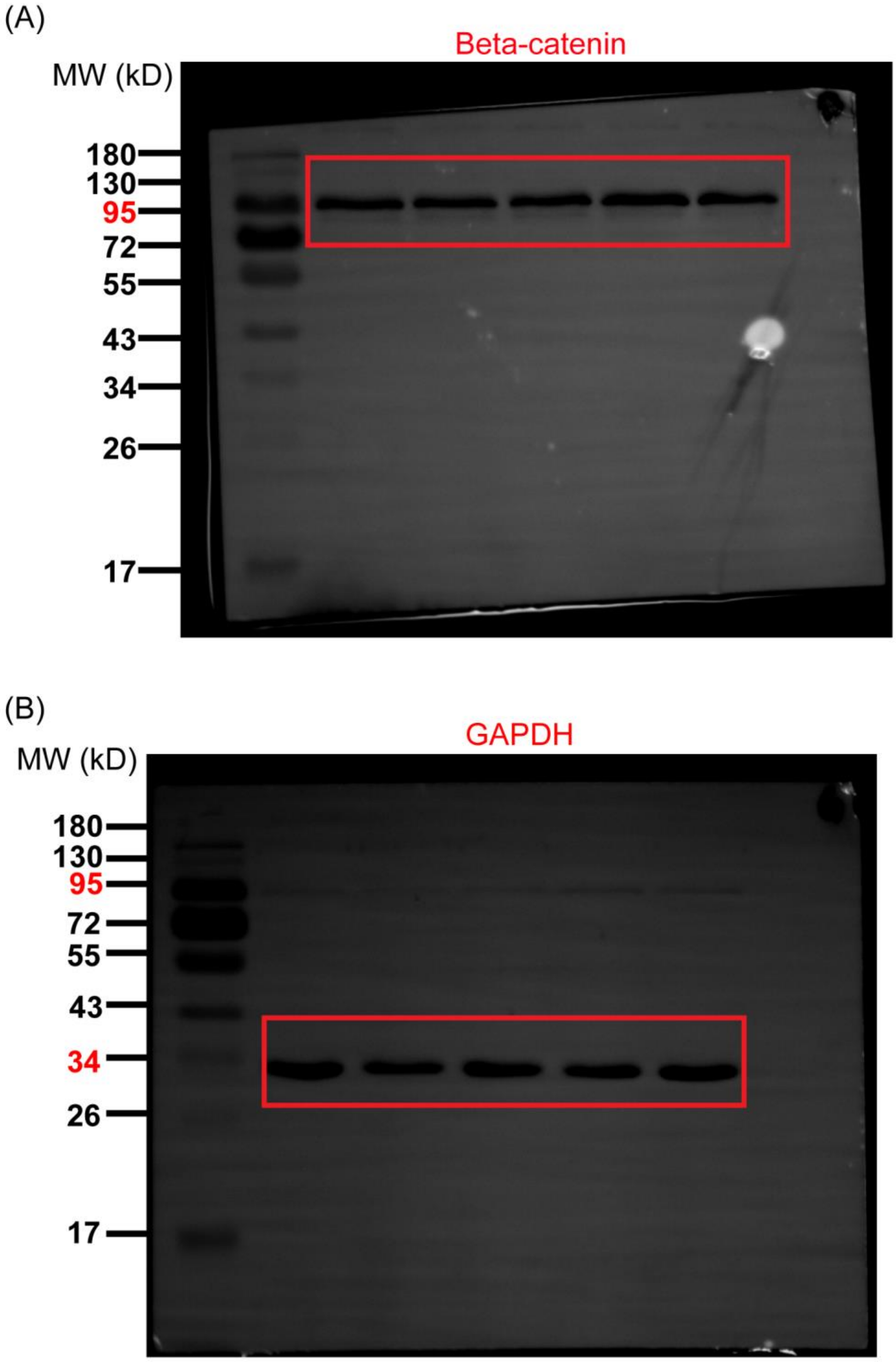


**Supplementary Figure 13. Uncropped western blot results shown in Supplementary Figure 11c.** Blots marked by red box were shown in Supplementary Figure 11c.

**Supplementary Table 1. *Sherlock* integrative analysis identifies SNPs associated with gene expression and schizophrenia simultaneously**

| **Gene symbol** | **LBF**[a] | ***P*-value**[b] | **Supporting SNP**[c] **(*cis* or *trans*)** | $P_{eQTL}$[d] | $P_{GWAS}$[e] | **Corrected *P***[f] |
|---|---|---|---|---|---|---|
| ***LOC376138*** | 9.38 | $1.11\times10^{-6}$ | rs4898494 (*trans*) | $3.08\times10^{-17}$ | $7.04\times10^{-6}$ | <0.05 |
| | | | rs902302(*trans*) | $7.95\times10^{-7}$ | $3.82\times10^{-5}$ | |
| ***KDELR1*** | 8.82 | $1.11\times10^{-6}$ | rs4735158 (*trans*) | $8.04\times10^{-6}$ | $3.33\times10^{-9}$ | <0.05 |
| | | | rs195204(*trans*) | $1.85\times10^{-6}$ | $2.48\times10^{-4}$ | |
| | | | rs16954069(*trans*) | $9.22\times10^{-6}$ | $8.33\times10^{-4}$ | |
| ***ALMS1*** | 7.63 | $1.11\times10^{-6}$ | rs6753344 (*cis*) | $1.37\times10^{-9}$ | $1.77\times10^{-6}$ | <0.05 |
| | | | rs11191393(*trans*) | $4.62\times10^{-6}$ | $1.15\times10^{-3}$ | |
| ***GLT8D1*** | 7.10 | $1.11\times10^{-6}$ | rs6795646 (*cis*) | $2.50\times10^{-8}$ | $7.15\times10^{-9}$ | <0.05 |
| ***ZNF323*** | 7.05 | $1.11\times10^{-6}$ | rs1150709 (*cis*) | $2.05\times10^{-8}$ | $7.05\times10^{-10}$ | <0.05 |
| ***CSNK2B*** | 6.70 | $1.11\times10^{-6}$ | rs2075800 (*cis*) | $5.39\times10^{-6}$ | $3.63\times10^{-11}$ | <0.05 |
| ***SLITRK5*** | 6.16 | $2.21\times10^{-6}$ | rs1265883 (*cis*) | $7.49\times10^{-6}$ | $5.04\times10^{-5}$ | <0.05 |
| | | | rs6465984 | $4.48\times10^{-6}$ | $1.41\times10^{-4}$ | |
| ***LOC375768*** | 5.93 | $2.21\times10^{-6}$ | rs9853627 (*trans*) | $4.85\times10^{-7}$ | $1.09\times10^{-6}$ | <0.05 |
| ***TBC1D15*** | 5.65 | $4.42\times10^{-6}$ | rs6582065 (*cis*) | $4.96\times10^{-6}$ | $5.12\times10^{-5}$ | <0.05 |
| ***ZNF446*** | 5.64 | $4.42\times10^{-6}$ | rs7012579 (*trans*) | $4.38\times10^{-7}$ | $1.51\times10^{-5}$ | <0.05 |
| | | | rs413019 | $8.04\times10^{-6}$ | $2.67\times10^{-4}$ | |

[a]LBF (the Logarithm of the Bayes Factor for the gene) is a critical indicator to assess if a specific gene is associated with schizophrenia through integrating the evidence from GWAS and brain eQTL. The larger of LBF is, the higher probability that the gene is associated with schizophrenia. For example, a LBF of 5.64 means that the interested gene is more likely (281 times, (*exp*(5.64) = 281)) to be associated with schizophrenia than the null hypothesis (no association). For a given gene, *Sherlock* first identifies all SNPs that are associated with the expression of this gene (these SNPs were called eSNPs). *Sherlock* then scores each eSNP based on the association significance between the eSNP and schizophrenia. A positive score would be assigned to the eSNP if this eSNP is also significantly associated with schizophrenia. A negative score would be assigned to the eSNP if this eSNP is not associated with schizophrenia. Disease-associations found only in the GWAS do not alter the score. For a given gene, individual LBF for each eSNP of this gene was calculated and the sum of the LBFs was used as the final LBF score of this gene. [b]*P* value derived from *Sherlock* statistical inferences. [c]SNPs associated with gene expression and schizophrenia simultaneously are listed. [d]*P* value from brain eQTL analysis[33]. [e]*P* value from GWAS of schizophrenia[5]. [f]*P* value was corrected by Bonferroni multiple testing correction.

**Supplementary Table 2. Schizophrenia risk genes identified by GWAS**

| | | | | *Gene Name* | | | | |
|---|---|---|---|---|---|---|---|---|
| *RCN3* | *ADAMTSL3* | *ZFYVE21* | *PCDHA5* | *GALNT10* | *THAP11* | *AC027228.1* | *ITIH3* | *GOLGA6L4* |
| *CREB3L1* | *DPYD* | *HCN1* | *PCDHA1* | *MSANTD2* | *DND1* | *IK* | *PAK6* | *ATG13* |
| *CYP17A1* | *ANKRD44* | *NDUFA6* | *ANP32E* | *TAC3* | *PCDHA6* | *ABCB9* | *MKL1* | *C4orf27* |
| *CYP26B1* | *GATAD2A* | *PPP1R16B* | *L3MBTL2* | *RAI1* | *ZEB2* | *STAC3* | *APH1A* | *FAM109B* |
| *OTUD7B* | *PCDHA7* | *PPP1R13B* | *CTRL* | *SGSM2* | *PSKH1* | *SRPK2* | *LUZP2* | *TSNAXIP1* |
| *KDM4A* | *PPP2R3A* | *R3HDM2* | *IGSF9B* | *TRIM26* | *AS3MT* | *SUGP1* | *MAN2A1* | *FANCL* |
| *IFT74* | *CDC25C* | *KCNV1* | *KCTD13* | *ZMAT2* | *ETF1* | *PLCL1* | *ZSWIM6* | *CNNM2* |
| *HAPLN4* | *PSMB10* | *AMBRA1* | *PCDHA9* | *GID4* | *ACTR5* | *TYW5* | *MED19* | *PCGF6* |
| *VPS14C* | *HIST1H2BL* | *SMDT1* | *CTNND1* | *TMX2* | *IREB2* | *C11orf31* | *SREBF1* | *EFHD1* |
| *PRSS16* | *CENPT* | *C16orf92* | *CHADL* | *TCF20* | *PTGIS* | *AGPHD1* | *PUS7* | *ZNF804A* |
| *NKAPL* | *SPCS1* | *AC073043.2* | *CR1L* | *LRRIQ3* | *C12orf79* | *HSPD1* | *CUL3* | *CSMD1* |
| *PSMA4* | *MYO18B* | *DGKZ* | *CHRNA3* | *TLE3* | *CLCN3* | *FAM57B* | *PODXL* | *MPP6* |
| *RILPL2* | *RFTN2* | *TMEM219* | *XRCC3* | *TSR1* | *PCDHA3* | *NCAN* | *PRRG2* | *ZDHHC5* |
| *TSPAN18* | *AC005477.1* | *NOSIP* | *SFXN2* | *FAM5B* | *DRG2* | *PARD6A* | *CKB* | *SREBF2* |
| *STT3A* | *FES* | *MIR137* | *ERCC4* | *F2* | *SLCO6A1* | *STAB1* | *SERPING1* | *STAT6* |
| *CHRM4* | *SMIM4* | *PLAA* | *NAB2* | *GRIA1* | *ATXN7* | *RANGAP1* | *NFATC3* | *MDK* |
| *SLC35G2* | *NUTF2* | *ATPAF2* | *ESAM* | *MAD1L1* | *NEK4* | *ADRBK2* | *NISCH* | *SBNO1* |
| *GNL3* | *SATB2* | *LRRC48* | *WBP2NL* | *LRP1* | *ACD* | *RERE* | *MMP16* | *PITPNM2* |
| *PTN* | *TMEM194A* | *SNAP91* | *NGEF* | *MAN2A2* | *DPP4* | *COQ10B* | *ZNF408* | *SHISA8* |
| *NXPH4* | *DPEP2* | *AKT3* | *SLC12A4* | *NEK1* | *CDK2AP1* | *CCDC68* | *CD46* | *BCL9* |
| *ITIH1* | *MSL2* | *HIST1H2BJ* | *LSM1* | *INA* | *PLEKHO1* | *MEF2C* | *ASPHD1* | *CNOT1* |
| *PGBD1* | *AL049840.1* | *NT5DC2* | *KCNB1* | *HARBI1* | *ZSCAN2* | *C1orf54* | *CLU* | *USMG5* |
| *INO80E* | *VRK2* | *REEP2* | *CTNNA1* | *DGKI* | *HSPA9* | *NOTCH4* | *SNX19* | *C2orf69* |
| *CA8* | *GRM3* | *TOM1L2* | *CYP2D6* | *DPEP3* | *ARHGAP1* | *SLC4A10* | *ZKSCAN4* | *GRIN2A* |
| *GFRA3* | *EP300* | *NAGA* | *OSBPL3* | *THOC7* | *ESRP2* | *DNAJC19* | *PSMD6* | *TBX6* |
| *NDUFA4L2* | *PCDHA10* | *TCF4* | *PCDHA8* | *TAF5* | *FAM53C* | *SETD8* | *ZNF536* | *PRKD1* |
| *CACNA1C* | *RENBP* | *TNFRSF13C* | *CA14* | *EPC2* | *PCDHA4* | *SRR* | *RIMS1* | *NRGN* |
| *TMCO6* | *ARL6IP4* | *CD14* | *MYO1A* | *MUSTN1* | *ANKRD63* | *MLL5* | *TRANK1* | *QPCT* |
| *PBX4* | *PDCD11* | *YPEL4* | *ENKD1* | *APOPT1* | *HARS2* | *MARS2* | *PCDHA2* | *SF3B1* |
| *DOC2A* | *GRAMD1B* | *IMMP2L* | *TRIM8* | *CHRNA5* | *MYO15A* | *GLT8D1* | *FXR1* | *SLC45A1* |
| *GFOD2* | *MIR548AJ2* | *HARS* | *PJA1* | *TAOK2* | *HIRIP3* | *FUT9* | *PBRM1* | *TM6SF2* |
| *WBP1L* | *SMG6* | *DDX28* | *CENPM* | *SEZ6L2* | *TBC1D5* | *C11orf87* | *ARL3* | *TMTC1* |
| *C12orf42* | *C12orf65* | *SDCCAG8* | *EPHX2* | *C1orf132* | *CNTN4* | *PLA2G15* | *CNKSR2* | *FONG* |
| *DUS2L* | *KLC1* | *C3orf49* | *SHMT2* | *CCDC39* | *SLC7A6OS* | *TSSK6* | *GPM6A* | *OGFOD2* |
| *C2orf82* | *NLGN4X* | *TMEM110* | *YPEL3* | *VSIG2* | *BOLL* | *GDPD3* | *ITIH4* | *TSNARE1* |
| *SLC7A6* | *RGS6* | *MUSTN1* | *VPS45* | *CKAP5* | *WHSC1L1* | *BRP44* | *C16orf86* | *AC005609.1* |
| *NRN1L* | *PLCB2* | *SCAF1* | *TRMT61A* | *HLA-DRB9* | *LCAT* | *C2orf47* | *BTBD18* | *BCL11B* |
| *NDUFA13* | *NT5C2* | *NCK1* | *CACNA1I* | *PRR12* | *PCGEM1* | *GIGYF2* | *DFNA5* | *ATP2A2* |
| *RANBP10* | *FURIN* | *TLE1* | *C10orf32* | *NDST3* | *WDR55* | *STAG1* | *PCCB* | *MAPK3* |
| *SLC39A8* | *BAG5* | *DRD2* | *EGR1* | *PTPRF* | *RRAS* | *CILP2* | *TMEM110* | |
| *NDUFA2* | *MAU2* | *MPHOSPH9* | *ALDOA* | *PLCH2* | *CACNB2* | *CHRNB4* | *KDM3B* | |
| *SLC38A7* | *EDC4* | *CLP1* | *HSPE1* | *PPP4C* | *KCNJ13* | *C1orf51* | *SLC32A1* | |

**Supplementary Table 3. Association significance between eSNPs of *ALMS1, CSNK2B, GLT8D1* and hippocampal structure in healthy subjects** [a]

| Gene symbol | LBF | *P*-value | Supporting SNP (*cis* or *trans*) | $P_{eQTL}$ | $P_{GWAS}$ | Hippocampal $P$[b] |
|---|---|---|---|---|---|---|
| *ALMS1* | 7.63 | $1.11\times10^{-6}$ | rs6753344 (*cis*) | $1.37\times10^{-9}$ | $1.77\times10^{-6}$ | **0.024** |
| *GLT8D1* | 7.10 | $1.11\times10^{-6}$ | rs6795646 (*cis*) | $2.50\times10^{-8}$ | $7.15\times10^{-9}$ | 0.83 |
| *CSNK2B* | 6.70 | $1.11\times10^{-6}$ | rs2075800 (*cis*) | $5.39\times10^{-6}$ | $3.63\times10^{-11}$ | 0.38 |

[a] Schizophrenia risk genes were identified by *Sherlock* integrative analysis, which integrates schizophrenia GWAS from PGC (N=35,476 cases and 46,839 controls[5]) and Brain eQTL data from Myers *et al.* (N=193)[6]. [b] Association significance between eSNP and hippocampal structure in ENIGMA sample (N=13,717)[7].

**Supplementary Table 4. Association significance between eSNPs of *ALMS1, CSNK2B, GLT8D1* and hippocampal structure in healthy subjects** [a]

| Gene symbol | LBF[a] | *P*-value | Supporting SNP (*cis* or *trans*) | $P_{eQTL}$[b] | $P_{GWAS}$ | Hippocampal $P$[b] |
|---|---|---|---|---|---|---|
| *ALMS1* | 6.34 | $3.25\times10^{-6}$ | rs56145559 (*cis*) | $2.11\times10^{-16}$ | $8.42\times10^{-8}$ | **0.026** |
| *GLT8D1* | 7.23 | $4.07\times10^{-7}$ | rs7620039 (*cis*) | $2.93\times10^{-9}$ | $3.47\times10^{-8}$ | 0.50 |
| *CSNK2B* | 6.15 | $5.69\times10^{-6}$ | rs144024892 (*cis*) | $8.49\times10^{-12}$ | $4.39\times10^{-11}$ | NA |

[a]Schizophrenia risk genes were identified by *Sherlock* integrative analysis, which integrates schizophrenia GWAS from PGC (N=35,476 cases and 46,839 controls)[5] and Brain eQTL data from Fromer *et al.* (N=467)[8]. [b]Association significance between eSNP and hippocampal structure in ENIGMA sample (N=13,717)[7]. NA, not available in ENIGMA sample.

**Supplementary Table 5. Association significance between eSNPs of *ALMS1, CSNK2B, GLT8D1* and hippocampal structure in healthy subjects** [a]

| Gene symbol | Top SNP (*cis* or *trans*) | $P_{eQTL}$ | $P_{GWAS}$ | $P_{SMR}$ | Hippocampal $P$[b] |
|---|---|---|---|---|---|
| *ALMS1* | rs7607892 (*cis*) | $1.54\times10^{-9}$ | $3.19\times10^{-6}$ | $2.22\times10^{-4}$ | **0.019** |
| *GLT8D1* | rs1961958 (*cis*) | $6.41\times10^{-27}$ | $2.41\times10^{-7}$ | $3.45\times10^{-6}$ | 0.38 |

[a] Schizophrenia risk genes were identified by *SMR* integrative analysis, which integrates schizophrenia GWAS from PGC (N=35,476 cases and 46,839 controls)[5] and Brain eQTL data from Myers *et al.* (N=193)[6]. [b] Association significance between eSNP and hippocampal structure in ENIGMA sample (N=13,717)[7].

**Supplementary Table 6. eSNPs of *ALMS1* gene are close to monomorphic in Han Chinese [a]**

| Gene symbol | eSNPs | Polymorphism | MAF in CHB | MAF in CEU | MAF in AFR |
|---|---|---|---|---|---|
| ***ALMS1*** | rs6753344 | G/A | **G: 0.005** | G: 0.217 | A: 0.398 |
| | rs56145559 | C/T | **T: 0.005** | T: 0.214 | C: 0.397 |
| | rs7607892 | C/T | **T: 0.005** | T: 0.215 | C: 0.409 |

[a] Frequency data were from the 1000 genomes project (http://www.internationalgenome.org/ )[9]. CHB - Han Chinese from Beijing; CEU-Europeans; AFR-Africans.

**Supplementary Table 7. eSNPs of *CSNK2B* and *GLT8D1* are associated with working memory (N=1,132)**

| Gene symbol | eSNPs | Allele | Task | Performance | P[a] |
|---|---|---|---|---|---|
| ***CSKN2B*** | rs2075800 | C/T | 3-Back | TT: 0.789; TC: 0.804; CC: 0.812 | **0.048** |
| ***GLT8D1*** | rs6795646 | C/T | 2-Back | CC: 0.904; CT: 0.905: TT: 0.893 | **0.049** |

[a]Association significance was assessed using linear regression model.

**Supplementary Table 8. Primer sequence for qPCR**

| Primer | Sequence |
|---|---|
| *Sox2*_F | 5’-CACAGATGCAACCGATGCA-3’ |
| *Sox2*_R | 5’-GGTGCCCTGCTGCGAGTA-3’ |
| *Klf4*_F | 5’-CACACAGGCGAGAAACCTTACC-3’ |
| *Klf4*_R | 5’-CGGAGCGGGCGAATTT-3’ |
| *Nanog*_F | 5’- AGGATGAAGTGCAAGCGGTG-3’ |
| *Nanog*_R | 5’- TGCTGAGCCCTTCTGAATCAG-3’ |
| *Nestin*_F | 5’-CCAGAGCTGGACTGGAACTC-3’ |
| *Nestin*_R | 5’-ACCTGCCTCTTTTGGTTCCT-3’ |
| *Gfap*_F | 5’-ACCAAATCCGTGTCAGAAGG-3’ |
| *Gfap*_R | 5’-CAGAAGGAAGGGAAGTGCTG-3’ |
| *Olig1*_F | 5’- GCAACTACATCCTGCTGCTG -3’ |
| *Olig1*_R | 5’- CACCAGCTGGGAGAGAGAAC -3’ |
| *Olig2*_F | 5’- CTGGTGTCTAGTCGCCCATC-3’ |
| *Olig2*_R | 5’- AGGAGGTGCTGGAGGAAGAT-3’ |
| *Neurod1*_F | 5’-CAAAGCCACGGATCAATCTT-3’ |
| *Neurod1*_R | 5’-CCCGGGAATAGTGAAACTGA-3’ |
| *Gapdh_F* | 5’-CTCAACTACATGGTCTACATGTTCCA-3’ |
| *Gapdh_R* | 5’-CCATTCTCGGCCTTGACTGT-3’ |

**References**


1 Benita Y, Cao Z, Giallourakis C, Li C, Gardet A, Xavier RJ. Gene enrichment profiles reveal T-cell development, differentiation, and lineage-specific transcription factors including ZBTB25 as a novel NF-AT repressor. *Blood* 2010; **115**: 5376-5384.

2 Kang HJ, Kawasawa YI, Cheng F, Zhu Y, Xu X, Li M *et al.* Spatio-temporal transcriptome of the human brain. *Nature* 2011; **478**: 483-489.

3 Iwamoto K, Bundo M, Kato T. Altered expression of mitochondria-related genes in postmortem brains of patients with bipolar disorder or schizophrenia, as revealed by large-scale DNA microarray analysis. *Hum Mol Genet* 2005; **14**: 241-253.

4 Kim S, Webster MJ. The stanley neuropathology consortium integrative database: a novel, web-based tool for exploring neuropathological markers in psychiatric disorders and the biological processes associated with abnormalities of those markers. *Neuropsychopharmacology* 2009; **35**: 473-482.

5 Schizophrenia Working Group of the Psychiatric Genomics Consortium*. Biological insights from 108 schizophrenia-associated genetic loci. *Nature* 2014; **511**: 421-427.

6 Myers AJ, Gibbs JR, Webster JA, Rohrer K, Zhao A, Marlowe L *et al.* A survey of genetic human cortical gene expression. *Nat Genet* 2007; **39**: 1494-1499.

7 Hibar DP, Stein JL, Renteria ME, Arias-Vasquez A, Desrivieres S, Jahanshad N *et al.* Common genetic variants influence human subcortical brain structures. *Nature* 2015; **520**: 224-229.

8 Fromer M, Roussos P, Sieberts SK, Johnson JS, Kavanagh DH, Perumal TM *et al.* Gene expression elucidates functional impact of polygenic risk for schizophrenia. *Nat Neurosci* 2016; **19**: 1442-1453.

9 Abecasis GR, Altshuler D, Auton A, Brooks LD, Durbin RM, Gibbs RA *et al.* A map of human genome variation from population-scale sequencing. *Nature* 2010; **467**: 1061-1073.
